# Effects of Mdivi-1 on Neural Mitochondrial Dysfunction and Mitochondria-Mediated Apoptosis in Ischemia-Reperfusion Injury After Stroke: A Systematic Review of Preclinical Studies

**DOI:** 10.3389/fnmol.2021.778569

**Published:** 2021-12-24

**Authors:** Nguyen Thanh Nhu, Qing Li, Yijie Liu, Jian Xu, Shu-Yun Xiao, Shin-Da Lee

**Affiliations:** ^1^Faculty of Medicine, Can Tho University of Medicine and Pharmacy, Can Tho, Vietnam; ^2^Department of Rehabilitation, Shanghai Xuhui Central Hospital/Zhongshan-Xuhui Hospital, Fudan University, Shanghai, China; ^3^Institute of Rehabilitation Medicine, Shanghai University of Traditional Chinese Medicine, Shanghai, China; ^4^Department of Brain and Mental Disease, Shanghai Hospital of Traditional Chinese Medicine, Shanghai, China; ^5^Department of Physical Therapy, Graduate Institute of Rehabilitation Science, China Medical University, Taichung, Taiwan; ^6^Department of Physical Therapy, Asia University, Taichung, Taiwan; ^7^School of Rehabilitation Medicine, Weifang Medical University, Weifang, China

**Keywords:** Mdivi-1, mitochondrial function, apoptosis, ischemia-reperfusion injury, stroke

## Abstract

This systematic review sought to determine the effects of Mitochondrial division inhibitor-1 (Mdivi-1) on neural mitochondrial dysfunction and neural mitochondria-mediated apoptosis in ischemia/reperfusion (I/R) injury after ischemic stroke. Pubmed, Web of Science, and EMBASE databases were searched through July 2021. The studies published in English language that mentioned the effects of Mdivi-1 on neural mitochondrial dysfunction and neural mitochondria-mediated apoptosis in I/R-induced brain injury were included. The CAMARADES checklist (for *in vivo* studies) and the TOXRTOOL checklist (for *in vitro* studies) were used for study quality evaluation. Twelve studies were included (median CAMARADES score = 6; TOXRTOOL scores ranging from 16 to 18). All studies investigated neural mitochondrial functions, providing that Mdivi-1 attenuated the mitochondrial membrane potential dissipation, ATP depletion, and complexes I-V abnormalities; enhanced mitochondrial biogenesis, as well as inactivated mitochondrial fission and mitophagy in I/R-induced brain injury. Ten studies analyzed neural mitochondria-mediated apoptosis, showing that Mdivi-1 decreased the levels of mitochondria-mediated proapoptotic factors (AIF, Bax, cytochrome *c*, caspase-9, and caspase-3) and enhanced the level of antiapoptotic factor (Bcl-2) against I/R-induced brain injury. The findings suggest that Mdivi-1 can protect neural mitochondrial functions, thereby attenuating neural mitochondria-mediated apoptosis in I/R-induced brain injury. Our review supports Mdivi-1 as a potential therapeutic compound to reduce brain damage in ischemic stroke (PROSPERO protocol registration ID: CRD42020205808).

**Systematic Review Registration:** [https://www.crd.york.ac.uk/prospero/], identifier [CRD42020205808].

## Introduction

Ischemic stroke is one of the most common diseases, causing a considerable number of deaths and disabilities globally (Roy-O’Reilly and McCullough, 2018). In brains suffering from ischemia and reperfusion (I/R) injury after ischemic stroke, neural cells are devastated, resulting in many neurologic deficits in stroke patients (Sekerdag et al., 2018; Andrabi et al., 2020). Numerous studies have been carried out to identify therapeutic methods that could reduce I/R-induced brain injury.

Neural mitochondrial dysfunction, characterized by neural mitochondrial respiratory deficiency and neural mitochondrial quality-control dysregulation, is considered the primary mechanism in I/R-induced brain injury (Andrabi et al., 2020; He et al., 2020; Carinci et al., 2021). In I/R neural cells, the mitochondrial respiratory chain (electron transport system), such as complexes I-IV, is impaired, leading to mitochondrial membrane potential (MMP) dissipation and ATP depletion (Galkin, 2019). In addition, neural mitochondrial quality-control dysregulation in I/R injury is exhibited by abnormal changes in mitochondrial biogenesis, dynamic (fusion/fission), and mitophagy (Anzell et al., 2018; Yang et al., 2018; He et al., 2020). Specifically, mitochondrial biogenesis regulators (e.g., PGC-1α, TFAM, and NRF-1) are increased in I/R neural cells in response to reductions in neural mitochondrial content (Yang et al., 2018). Neural mitochondrial fission has been shown to be augmented, increasing the number of dysfunctional mitochondria in I/R neurons (Anzell et al., 2018; Yang et al., 2018). Furthermore, research has suggested that changes in neural mitophagy (autophagy of neural mitochondria) are varied after ischemic stroke, playing both positive and negative roles in the brain under I/R injury (Anzell et al., 2018).

Neural mitochondrial dysfunction has been shown to promote neural apoptosis in ischemic stroke (Sekerdag et al., 2018; Andrabi et al., 2020). In brains with I/R injury, the levels of B-cell lymphoma (Bcl-2) proapoptotic factors (e.g., Bax, Bad, and tBid) are increased, which bind and inactivate Bcl-2 antiapoptotic factors (e.g., Bcl-2, Bcl-xl, and Bcl-w) (Ouyang and Giffard, 2014; Li et al., 2016). Consequently, the mitochondrial permeability transition pore (mPTP) is prompted to open, releasing apoptosis-inducing factor (AIF), endonuclease G (EndoG), and cytochrome *c* from neural mitochondria to the neural cytosol (Carinci et al., 2021). Cytosolic AIF and EndoG in turn directly transfer to the neural nucleus to damage DNA and cause neural mitochondria-mediated caspase-independent apoptosis (Zhang et al., 2007; Uzdensky, 2019). In addition, once released, cytosolic cytochrome *c* forms the apoptosome, activating caspase-9 and then caspase-3 to induce neural mitochondria-mediated caspase-dependent apoptosis (Broughton et al., 2009). The strong association between neural mitochondrial dysfunction and neural mitochondria-mediated apoptosis has recently been considered a potential target of studies for therapy in ischemic stroke (Carinci et al., 2021).

Mitochondrial division inhibitor-1 (Mdivi-1), a cellular-permeable small molecule, has emerged as a promising therapeutic compound that reduces mitochondrial dysfunction and apoptosis in myocardial infarction and neurodegenerative diseases (Cassidy-Stone et al., 2008). Previous evidence has reported that Mdivi-1 could attenuate mitochondrial fission and mitochondrial outer membrane permeabilization, reducing mitochondria-mediated apoptosis (Cassidy-Stone et al., 2008; Duan et al., 2020). In ischemic stroke, a study showed that Mdivi-1 could increase neural mitochondrial respiratory function and decrease the expression of apoptotic factors (e.g., Bax) in I/R hippocampal neurons (Li et al., 2016). Another study showed that Mvidi-1 treatment increased cellular ATP production and reduced neural apoptotic cells in ischemic stroke models both *in vivo* and *in vitro* (Zhao et al., 2014). Additionally, Mdivi-1 has been reported to recover mitochondrial complex I activity, thereby attenuating mitochondrial dysfunction in I/R cortical neurons *in vitro* (Bordt et al., 2017). In order to comprehensively understand the neuroprotective effects of Mdivi-1 on ischemic stroke, our review was carried out to systematically summarize the evidence from both *in vivo* and *in vitro* studies, focusing on two goals: (1) the effects of Mdivi-1 on neural mitochondrial dysfunction in I/R-induced brain injury after ischemic stroke, and (2) the effects of Mdivi-1 on neural mitochondria-mediated apoptosis in I/R-induced brain injury after ischemic stroke.

## Methods

### Protocol and Registration

The protocol of this review was registered on the “International Prospective Register of Systematic Review” (PROSPERO), with the registration number CRD42020205808. We followed the “Preferred Reporting Items for Systematic Reviews and Meta-Analyses” (PRISMA) guideline (2020) (Page et al., 2021) for conducting and writing the review.

### Eligibility Criteria

#### Types of Study Designs

Preclinical studies (*in vivo* and *in vitro* studies) that were published in English language without any limitations of publication time. For *in vivo* studies, experimental studies had to include separate animal groups. For *in vitro* studies, the studies needed separated groups of neural cells. Single group studies, cross-sectional studies, protocol studies, conference abstracts, and reviews were excluded.

#### Types of Models

For *in vivo* models, ischemic stroke animal models were included (all species, age, and sex). Animal models that combined stroke with other injuries in the brain were excluded. For *in vitro* models, neural cell models that mimic I/R injury were included. Neural cell models that combined ischemic injury and other conditions were also excluded.

#### Types of Intervention

For *in vivo* models, the intervention required mitochondrial division inhibitor 1 (Mdivi-1) administration. Similarly, for *in vitro* models, neural cells had to be treated with Mdivi-1. Data on dosage and timing injection needed to be available. The studies that combined Mdivi-1 and other therapies was excluded.

#### Type of Comparators

For *in vivo* models, the comparators had to be ischemic stroke animals that did not receive any treatments. For *in vitro* models, the comparators had to be neural cells that were cultured to mimic I/R injury without any treatments. Studies that did not have untreated ischemic stroke models (both *in vivo* and *in vitro* models) were excluded.

#### Type of Outcomes

For the effects of Mdivi-1 on neural mitochondrial functions in I/R injury (the first outcome), data on mitochondrial respiratory function and mitochondrial quality-control (biogenesis, dynamics, and mitophagy) were included. The neural mitochondrial respiratory function was evaluated through electron transport system activities, mitochondrial membrane potential, ATP level, reactive oxidative species, and antioxidant production. The neural mitochondrial quality-control was evaluated through the levels of mitochondrial DNA, mitochondrial biogenesis regulators, mitochondrial fusion factors, mitochondrial fission factors, mitophagy related-proteins, and mitochondrial number. For the effects of Mdivi-1 on neural mitochondria-mediated apoptosis in I/R injury (the second outcome), data on the alterations of apoptotic cell number, DNA fragmentation, mitochondria-mediated proapoptotic factors, and mitochondria-mediated antiapoptotic factors were included.

### Data Sources and Search Strategy

Eligible papers were searched by keywords on PubMed, Web of Science, and EMBASE databases through July 2021, with a combination of the following terms: *(“ischemia” OR “ischemic” OR “stroke” OR “cerebral vascular accident”) AND (“Mdivi-1” OR “Mitochondrial division inhibitor-1” OR “fission inhibition”) AND (“mitochondria” OR “mitochondrial” OR “mitochondrion” OR “mitophagy” OR “brain” OR “neuron” OR “neural apoptosis” OR “neural cell death”)*. In brief, the titles and abstracts of studies were read to exclude duplicates and irrelevant studies that did not provide the required information of ischemic stroke and Mdivi-1 in their abstracts. Then the full texts were read all and the studies were selected based on eligibility criteria. Two independent evaluators carried out the study selection procedure. When disagreement occurred, two evaluators discussed with a third consultant to make the final decision.

### Data Collection Process

The text, graphs, and tables of reviewed studies were read to extract data, including study characteristics and outcomes. If required data were not mentioned in the included papers, the corresponding authors were contacted for requests. The process of data extraction was conducted by two independent reviewers. For study characteristics, we extracted the data of the first author’s name, published year, models (for *in vivo* models: type, species, sex; for *in vitro* models: cell type and cell models), and Mdivi-1 treatment (dosage and timing). For the outcome extraction, we extracted and grouped data into two parts: (1) the effects of Mdivi-1 on neural mitochondrial dysfunction, including neural mitochondrial respiratory function and neural mitochondrial quality-control in I/R-induced brain injury after ischemic stroke; and (2) the effects of Mdivi-1 on the neural mitochondria-mediated apoptosis in I/R-induced brain injury after ischemic stroke.

### Study Quality Evaluation

The quality of *in vivo* studies was evaluated using the “Collaborative Approach to Meta-Analysis and Review of Animal Data from Experimental Studies” (CAMARADES) checklist with 10 items (Auboire et al., 2018). The predesigned TOXRTOOL quality checklist was used to evaluate the quality of *in vitro* studies (Schneider et al., 2009). Two authors independently evaluated and filled in the predesigned datasheets of the CARAMADES checklist and TOXRTOOL checklist. Then, two results were compared, and the differences were discussed between two examiners and a third consultant.

### Data Synthesis and Presentation

The results of the search procedure were reported by the PRISMA flowchart (2020 version) and the narrative synthesis. We used the table and text to provide the data on study characteristics and outcomes. For presentation of study characteristics, the summaries of study designs, ischemic stroke models, Mvidi-1 treatment, and types of outcomes were provided. For presentation of outcomes, the effects of Mvidi-1 treatment on neural mitochondrial dysfunction and neural mitochondria-mediated apoptosis in I/R injury after ischemic stroke were described. In addition, the methodology quality of studies and the risk of bias were synthesized in two predesigned tables (used for *in vivo* or *in vitro* studies).

## Results

### Search Results

**FIGURE 1:**
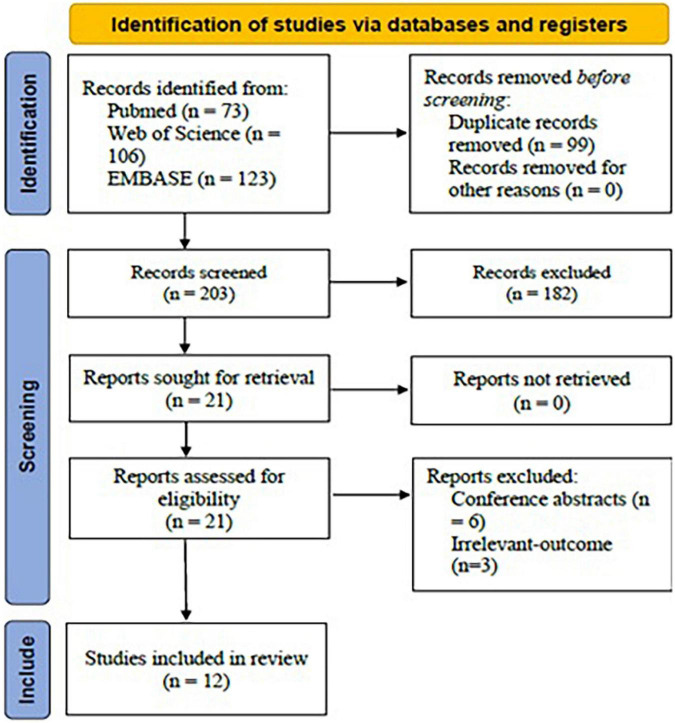
PRISMA flow chart for study selection protocol (2020 version).

Using the mentioned term formula, 302 potential articles were found from PubMed (*n* = 73), Web of Science (*n* = 106), and EMBASE (*n* = 123). Then, 99 duplicates were removed. After title and abstract screening, 182 irrelevant-topic studies were removed. After full-text review, 9 studies were excluded, including 06 conference abstracts and 03 studies irrelevant-outcome studies. Finally, 12 studies were included in the current systematic review for analysis (Figure 1).

### Study Characteristics

#### Type of Study Design

The included studies consisted of *in vivo* studies (*n* = 7), *in vitro* studies (*n* = 4) and a study conducted both *in vivo* and *in vitro* (*n* = 1).

#### Type of Ischemic Models

For the *in vivo* studies, rats and mice were used. Four included studies used the MCAO models, three studies used cardiac arrest/cardiopulmonary resuscitation models, and the other study used a transient global ischemia model. For the *in vitro* studies, the cell models were oxygen-glucose deprivation models with hippocampal neurons (*n* = 2), N2a cells (*n* = 1), SH-SY-5Y cells (*n* = 1), or E18 rat cortical neurons (*n* = 1). Those models are commonly used as ischemic stroke models with I/R injury.

#### Type of Mdivi-1 Treatment

The *in vivo* studies used a variety of Mvidi-1 dosages, ranging from 0.24 to 20 mg/kg. The *in vitro* studies used five different dosages of Mdivi-1 (5 μM, 10 μM, 25 μM, 50 μM, and 50 mM) to treat the cell models. Six studies conducted pretreatment, and six studies conducted post-treatment.

#### Type of Outcomes

All studies investigated the effects of Mdivi-1 on neural mitochondrial dysfunction, including mitochondrial respiratory function and mitochondrial quality-control (biogenesis, fusion/fission, and mitophagy). For neural mitochondrial respiratory function, the studies reported data on complex I-V activities, mitochondrial membrane potential (MMP), ATP, reactive oxygen species (ROS), and superoxide dismutase (SOD). For neural mitochondrial biogenesis, the studies reported data on the level of mitochondrial DNA and mitochondrial biogenesis regulators, i.e., peroxisome proliferator-activated receptor gamma coactivator 1-alpha (PGC-1α), mitochondrial transcription factor A (TFAM), and nuclear respiratory factor 1 (NRF-1). For neural mitochondrial fusion/fission, the studies reported data on dynamin-related protein-1 (Drp-1), dynamin-like 120 kDa protein (Opa1), and mitofusin-1 (Mfn1). For neural mitophagy, the studies reported data on mitochondria-related factors, i.e., PTEN-induced kinase-1 (PINK1) and Parkin, as well as mitochondria number. Ten studies analyzed the effects of Mdivi-1 on the neural mitochondrial-mediated apoptosis, reporting the percentage of TUNEL positive cells and the expressions of several apoptotic factors, i.e., AIF, Bax, Bcl-2, cytochrome *c*, and caspase-3. In addition, those studies also mentioned the alterations of DNA fragmentation (Table 1).

**TABLE 1:** Characteristics of the included studies.

Study	Model	Experimental groups	Intervention	Outcomes			
				Neural mitochondrial respiratory function	Neural mitochondrial biogenesis	Neural mitochondrial fusion/fission and mitophagy	Neural mitochondria-mediated apoptosis
Zhang et al., 2013—*in vivo* study	Male Wistar rats with MCAO model (using ischemic tissue from the chiasma opticum to 4 mm posterior)	20 rats/group, four groups 1. MCAO rats 2. MCAO rats (vehicle) 3. MCAO rats + Mdivi-1 (dose 1) 4. MCAO rats + Mdivi-1 (dose 2)	Mdivi-1 dose 1 (1.2 mg/kg) and dose 2 (0.24 mg/kg), pre-treatment			Dose 1: ↓Drp-1 protein and mRNA levels Dose 2: No changes	Dose 1: ↓TUNEL apoptotic activity ↓cytochrome *c* protein and mRNA levels Dose 2: no changes
Ma et al., 2016—*in vivo* study	Male Wistar rats with MCAO model (using ischemic cortex tissue)	10 rats/group 1. Normal rats 2. MCAO rats 3. MCAO rats + Mdivi-1	Mdivi-1 (1 mg/kg, i.p.), post-treatment		↑mRNA levels of PGC-1α, NRF-1, and TFAM.	↓ mitochondrial fragmentation. ↓mitochondrial Drp-1 protein level No change cytosolic Opa-1 protein level	↓TUNEL apoptotic activity ↓ cytosolic cytochrome *c* protein level.
Li et al., 2015—*in vivo* study	Male Sprague–Dawley rats with CA/CRP models (using hippocampal CA1 region for TUNEL assay; using total ischemic hemisphere tissue for Western blot)	50 rats, four groups 1. Vehicle (*n* = 8) 2. CA/CPR rats (*n* = 14) 3. CA/CPR rats + Mdivi-1 dose 1 (*n* = 14) 4. CA/CPR rats + Mdivi-1 dose 2 (*n* = 14)	Mdivi-1 dose 1 (1.2 mg/kg) and dose 2 (0.24 mg/kg), post-treatment			Dose 1: ↓mitochondrial Drp-1 protein level Dose 2: No changes	Dose 1: ↓TUNEL ↓cytosolic cytochrome *c*, AIF, caspase-3 protein levels Dose 2: No significant changes
Cui et al., 2016—*in vivo* study	Male C57BL/6 mice MCAO model (using brain tissue from ischemic striatum)	9–11 rats/group, three groups 1. Sham 2. MCAO rats 3. MCAO rats + Mdivi-1	Mdivi-1 (20 mg/kg) i.p; pre-treatment	↓ the release of ATP from neural mitochondria			
Chuang et al., 2016—*in vivo* study	Male Sprague-Dawley rats with TGI model (using ischemic hippocampal tissue)	4–6 rats/group, three groups 1. TGI rats 2. Vehicle TGI rats 3. TGI rats + Mdivi-1	Mdivi-1 (2.4 mg/kg), pre-treatment			↓p-Drp-1 (Ser616) protein level	↓ DNA fragmentation ↓ caspase-3 protein level
Wang et al., 2018—*in vivo*	Male Sprague-Dawley rats with CA/CRP models (using ischemic hippocampal tissue)	146 rats, four groups: 1. Normal rats (*n* = 41) 2. CA/CRP rats (*n* = 12) 3. CA/CRP rats + Mdivi-1 (*n* = 39) 4. CA/CRP rats + Hypothermic (*n* = 38)	1.2 mg/kg of mdivi-1, intravenously, post-treatment	↑ MMP, ATP ↓ ROS level (in mitochondria)			↓ TUNEL positive cells
Huang et al., 2021—*in vivo* study	Male Sprague–Dawley rats with CA/CRP model (using ischemic hippocampal tissue)	100 rats, 4 group: 1. Normal rats. 2. CA/CRP rats 3. CA/CRP rats + Mdivi-1 4. CA/CRP rats + remote ischemic post-conditioning	1.2 mg/kg of mdivi-1, intravenously, post-treatment	↑ MMP	↑mitochondrial DNA level	↑mitochondrial fusion factor: Mfn1 protein level ↓mitophagy: PINK1 and Parkin protein levels	↓ TUNEL positive cells
Zhao et al., 2014—both *in vivo* and *in vitro* study	*In vivo*: Male MCAO mice model (using ischemic cortex tissue) *In vitro*: SH−SY−5Y cells with OGD model	*In vivo*: (6–9/group) 1. Sham 2. Sham + Mdivi-1 3. MCAO mice 4. MCAO mice + Mdivi-1 *In vitro*: 1. Normal cells 2. Normal cells + Mdivi-1 3. OGD cells 4. OGD cells + Mdivi-1	*In vivo*: Mdivi−1 (20 mg/kg), pre-treatment *In vitro*: mdivi-1 (10 μM), pre-treatment	*In vivo* ↓ ATP. *In vitro* ↑ MMP, ATP		↓ *in vivo* mitochondrial fragmentation ↓*in vitro* mitochondrial fragmentation	*In vivo* ↓ TUNEL and the protein level of cytosolic cytochrome *c*. *In vitro* ↑ cell viability Block Bax insertion and oligomerization ↓ cytosolic cytochrome *c*
Li et al., 2016—*in vitro* study	Hippocampal neurons with OGD model	1. Control 2. Vehicle 3. OGD cells 4. OGD cells + Mdivi-1	mdivi-1 (50 mM), post-treatment	↑ MMP ↑ complex I-IV activities and ↑ATP level		↓Drp-1 protein level	↓ Bax protein level ↑ Bcl-2 protein level
Wang et al., 2014—*in vitro* study	Hippocampal cells with OGD model	1. Control 2. Vehicle 3. OGD cells 4. OGD cells + Mdivi-1	mdivi-1 (50 μM), pre-treatment	↓ ROS, ↑ SOD		↓Drp-1 protein level	↓ apoptotic cells ↓Bax and cytochrome *c* protein level ↑ the protein level of Bcl-2
Zhou et al., 2017—*in vitro* study	N2a cells with OGD model	1. Normal cells 2. OGD cells 3. OGD cells + Mdivi-1	Mdivi-1 (5 μM), pre-treatment	↓ mPTP opening, ↑ MMP		↓Drp-1 protein level	↓Bax, cytosolic cytochrome *c*, caspase-3, and caspase-9 protein levels ↓ apoptotic cells.
Zhang et al., 2014—*in vitro*	E18 rats cortical neurons with OGD model	1. Normal cells 2. OGD cells 3. OGD cells + Mdivi-1	Mdivi-1 25 μM; post-treatment			Maintain neural mitochondria number	

*MCAO, middle cerebral artery occlusion; OGD, Oxygen-glucose deprivation; TGI, transient global ischemia; CA/CRP, cardiac arrest/cardiopulmonary resuscitation; i.p., Intraperitoneal injection; MMP, mitochondrial membrane potential; mPTP, mitochondrial permeability transition pore; ROS, reactive oxygen species; SOD, superoxide dismutase; PGC-1α, peroxisome proliferator-activated receptor gamma coactivator 1-alpha; NRF-1,2, nuclear respiratory factor 1 and 2; TFAM, mitochondrial transcription factor A; Drp-1, Dynamin-related protein-1; Opa1, Dynamin-like 120 kDa protein; AIF, apoptosis-inducing factor.*

### Outcome Summary

#### The Effects of Mdivi-1 on Neural Mitochondrial Dysfunction in I/R-Induced Brain Injury After Ischemic Stroke

Seven studies evaluated the effects of Mdivi-1 treatment on neural mitochondrial respiratory function in I/R-induced brain injury after ischemic stroke (Wang et al., 2014, 2018; Zhao et al., 2014; Cui et al., 2016; Li et al., 2016; Zhou et al., 2017; Huang et al., 2021). The activities of neural mitochondrial complexes I, II, III, and IV were shown to be suppressed in I/R injury, whereas those activities were significantly activated by Mdivi-1 treatment (Li et al., 2016). Five studies showed that neural mitochondrial membrane potential (MMP) was reduced in I/R injury compared to normal, whereas this level was significantly enhanced by Mdivi-1 treatment (Zhao et al., 2014; Li et al., 2016; Zhou et al., 2017; Wang et al., 2018; Huang et al., 2021). Three studies reported that ATP levels were reduced in I/R injury compared to controls, whereas treatment with Mdivi-1 significantly restored ATP levels in I/R injury (Zhao et al., 2014; Li et al., 2016; Wang et al., 2018). In addition, another study showed that Mdivi-1 treatment reduced the release of ATP from neural mitochondria to extracellular spaces (Cui et al., 2016). Furthermore, other studies showed that Mdivi-1 treatment could reduce neural mitochondrial ROS levels as well as increase SOD levels in I/R injury, suggesting that Mdivi-1 could attenuate the neural mitochondrial respiratory deficiency in I/R-induced brain injury after ischemic stroke (Wang et al., 2014, 2018).

Two studies investigated the effects of Mdivi-1 on neural mitochondrial biogenesis in I/R injury after ischemic stroke (Ma et al., 2016; Huang et al., 2021). One of those showed that the protein levels of neural mitochondrial biogenesis regulators (PGC-1α, TFAM, and NRF-1) were significantly increased in I/R injury compared to normal tissue, and these levels were further upregulated by Mdivi-1 treatment (Ma et al., 2016). The other study provided that the level of neural mitochondrial DNA was decreased in I/R injury compared to controls, whereas Mdivi-1 treatment enhanced this level against I/R injury (Huang et al., 2021).

Seven studies analyzed the effects of Mdivi-1 on neural mitochondrial fission, providing that Mdivi-1 treatment reduced the mRNA and protein levels of fission promotor—Drp-1 in I/R injury (Zhang et al., 2013; Wang et al., 2014; Li et al., 2015, 2016; Chuang et al., 2016; Ma et al., 2016; Zhou et al., 2017). Supportively, Mdivi-1 has reportedly reduced the mitochondrial fragmentation in I/R neural cells both *in vivo* and *in vitro* (Zhao et al., 2014; Ma et al., 2016). Regarding neural mitochondrial fusion, one study provided that Mdivi-1 had no effect on the protein level of neural inner-membrane mitochondrial fusion factor (Opa1) in I/R injury (Ma et al., 2016). In contrast, another study showed that Mdivi-1 increased the protein level of neural outer-membrane mitochondrial fusion factor (Mfn1) (Huang et al., 2021).

Two studies evaluated the effects of Mdivi-1 on neural mitophagy in I/R injury after ischemic stroke (Zhang et al., 2014; Huang et al., 2021). One study showed that the protein levels of mitophagy-related proteins (i.e., PINK1 and Parkin) in neural mitochondria were increased in I/R injury compared to controls, whereas these levels were reduced by Mdivi-1 treatment (Huang et al., 2021). Another study showed that the number of neural mitochondria in I/R neural cells treated by Mdivi-1 was significantly higher than that in the same kind of cells without treatment (Zhang et al., 2014).

#### The Effects of Mdivi-1 on Mitochondria-Mediated Neural Apoptosis in I/R-Induced Brain Injury After Ischemic Stroke

To summary the effects of Mdivi-1 on mitochondria-mediated neural apoptosis in I/R-induced brain injury after ischemic stroke, the findings from 10 relevant studies were included (Zhang et al., 2013; Wang et al., 2014, 2018; Zhao et al., 2014; Li et al., 2015, 2016; Chuang et al., 2016; Ma et al., 2016; Zhou et al., 2017; Huang et al., 2021). Eight studies provided that neural apoptotic cells increased significantly in I/R injury compared to normal cells, whereas Mdivi-1 treatment reduced this level in I/R injury both *in vivo* (Zhang et al., 2013; Li et al., 2015; Ma et al., 2016; Wang et al., 2018; Huang et al., 2021) and *in vitro* (Wang et al., 2014; Zhao et al., 2014; Zhou et al., 2017). Regarding the proapoptotic factors, three studies showed that the protein levels of Bax were increased in I/R hippocampal cells compared to normal cells, whereas these levels were reduced in I/R injury with Mdivi-1 treatment (Wang et al., 2014; Li et al., 2016; Zhou et al., 2017). Six studies showed that the protein levels of the downstream components of mitochondria-mediated caspase-dependent apoptotic pathways, including cytochrome *c* (Zhang et al., 2013; Wang et al., 2014; Li et al., 2015; Ma et al., 2016; Zhou et al., 2017), caspase-9 (Zhou et al., 2017), and caspase-3 (Zhang et al., 2013; Zhao et al., 2014; Li et al., 2015; Zhou et al., 2017), were augmented in I/R injury compared to controls, whereas these levels were significantly reduced by Mdivi-1 treatment. Furthermore, AIF, a representative of neural mitochondria-mediated caspase-independent apoptotic factors, was observed to be increased in I/R injury compared to normal, and this level was reduced by Mdivi-1 treatment (Li et al., 2015). Consistently, one study showed that DNA fragmentation increased in I/R injury without any treatment, whereas this level reduced by Mdivi-1 treatment (Chuang et al., 2016). In addition, two studies provided that Mdivi-1 treatment could attenuate the I/R-suppressed protein level of Bcl-2 (anti-apoptotic factor) (Wang et al., 2014; Li et al., 2016).

### Methodology Quality of the Included Studies

Regarding *in vivo* studies, the median CAMARADES score was 6. All studies were conducted with the appropriate models of I/R-induced brain injury and clearly provided anesthetics procedures. Moreover, all studies stated that they complied with available regulatory requirements and declared no conflicts of interest. Six studies (75%) reported data on the control temperature. Six studies (75%) randomized the experimental groups. However, none of the studies carried out allocation concealment, and only one study conducted the blinded outcome assessment. The sample size was also not calculated by all studies (Table 2).

**TABLE 2:** The quality of *in vivo* studies basing-on the CAMARADES checklist.

Author	CAMARADES checklist of study quality										
	1	2	3	4	5	6	7	8	9	10	Total
Zhang et al., 2013	✓	✓	✓			✓	✓		✓		6
Ma et al., 2016	✓	✓				✓	✓	✓	✓	✓	7
Li et al., 2015	✓	✓	✓			✓	✓		✓		6
Cui et al., 2016	✓		✓			✓	✓		✓	✓	6
Chuang et al., 2016	✓	✓				✓	✓		✓	✓	6
Wang et al., 2018	✓	✓	✓			✓	✓		✓	✓	7
Huang et al., 2021	✓	✓	✓			✓	✓		✓	✓	7
Zhao et al., 2014 (*in vivo*)	✓					✓	✓		✓	✓	5

*(1) Publication in peer-reviewed journal, (2) statement of control of temperature, (3) randomization of treatment or control, (4) allocation concealment, (5) blinded assessment of out-come, (6) avoidance of anesthetics with marked intrinsic properties, (7) use of animals with ischemia-reperfusion brain injury, (8) sample size calculation, (9) statement of compliance with regulatory requirements, (10) statement regarding possible conflict of interest.*

For *in vitro* studies, according to the TOXRTOOL checklist, two studies reached a sore of 18, 1 study reached a score of 17, and 2 study reached a score of 16. Moreover, all studies met all red items (the important items in the TOXRTOOL checklist). Therefore, the evidence from these studies were considered to be reliable without restrictions (Table 3).

**TABLE 3:** The quality of *in vitro* studies basing-on the ToxRTool checklist.

Author	TOXRTOOL checklist of study quality																			
	1	2	3	4	5	6	7	8	9	10	11	12	13	14	15	16	17	18	Total	Reliability of evidence
Zhao et al., 2014	1	1	1	1	1	1	1	1	1	1	1	1	1	1	1	1	1	1	18	Reliability without restrictions
Li et al., 2016	1	0	0	1	1	1	1	1	1	1	1	1	1	1	1	1	1	1	16	Reliability without restrictions
Wang et al., 2014	1	1	1	1	1	1	1	1	1	1	1	1	1	1	1	1	1	1	18	Reliability without restrictions
Zhou et al., 2017	1	0	1	1	1	1	1	1	1	1	1	1	1	1	1	1	1	1	17	Reliability without restrictions
Zhang et al., 2014	1	0	0	1	1	1	1	1	1	1	1	1	1	1	1	1	1	1	16	Reliability without restrictions

*(1) Test substance identification; (2) substance purity statement; (3) the source/origin information of the substance; (4) information on physicochemical properties of the test item given; (5) cell culture description; (6) the source/origin of cell culture; (7) necessary information on cell culture properties, conditions of cultivation and maintenance; (8) the method of Mdivi-1 administration; (9) doses or concentration statement; (10) frequency and duration of exposure as well as time-points of observations statement; (11) have negative controls; (12) have positive controls; (13) the number of replicates; (14) are the study endpoint(s) and their method(s) of determination clearly described?; (15) is the description of the study results for all endpoints investigated transparent and complete?; (16) are the statistical methods for data analysis given and applied in a transparent manner?; (17) is the study design chosen appropriate for obtaining the substance-specific data aimed at?; (18) are the quantitative study results reliable? The red items include items 1, 9, 10, 11, 12, and 17.*

## Discussion

### Summary of Evidence

**FIGURE 2:**
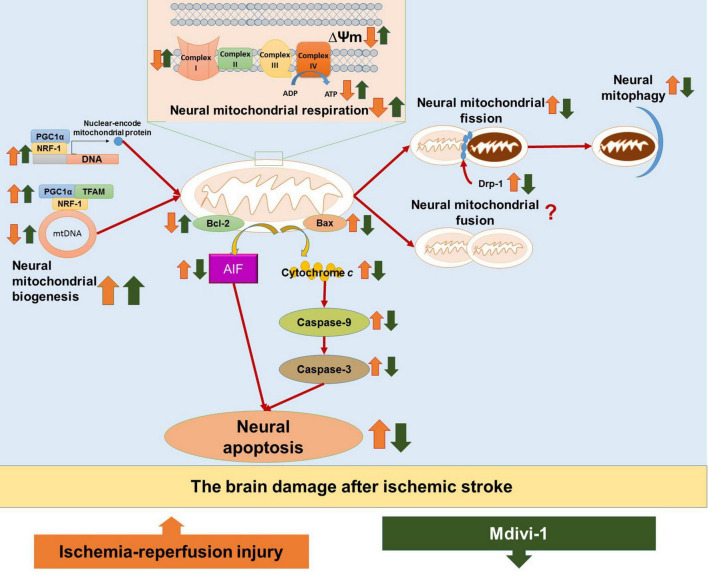
The hypothesized diagram. The figure provides the mechanisms of ischemia/reperfusion (I/R) injury in the brain after ischemic stroke, as well as the therapeutic effects of Mdivi-1 on neural mitochondria functions and neural mitochondria-mediated apoptosis. I/R injury is shown to induce neural mitochondrial respiration deficiency, as evidenced by decreases in mitochondrial membrane potential (ΔΨm), ATP production, and neural mitochondrial complexes I–IV. In addition, I/R injury could dysregulate neural mitochondrial quality-control, as evidenced by increases in neural mitochondrial biogenesis regulators (e.g., PGC-1α, TFAM, and NRF-1) as a compensatory response to the reduction of mitochondrial content including mitochondrial DNA (mtDNA), as well as increases in mitochondrial fission (Drp-1) and neural mitophagy. Neural mitochondrial respiratory deficiency and neural mitochondrial quality-control dysregulation in I/R injury promote neural mitochondria-mediated apoptosis, as evidenced by the activation of apoptotic factors, including mitochondria-mediated caspase-independent apoptotic factors (e.g., AIF), mitochondria-mediated caspase-dependent upstream proapoptotic factors (e.g., Bax), and mitochondria-mediated caspase-dependent upstream proapoptotic factors (e.g., cytochrome *c*, caspase-9, and caspase-3), as well as the inactivation of antiapoptotic factors (e.g., Bcl-2). The included studies suggest that Mdivi-1 could restore mitochondrial membrane potential (ΔΨm), enhance ATP production and normalize neural mitochondrial complexes I-V, suggesting that Mdivi-1 could attenuate neural mitochondrial respiratory deficiency against I/R injury after ischemic stroke. In addition, Mdivi-1 has been shown to further enhance biogenesis regulators (e.g., PGC-1α, TFAM, and NRF-1) to increase mtDNA, inactivate mitochondrial fission factor (Drp-1), and suppress neural mitophagy, implying that Mdivi-1 could protect neural mitochondria quality-control against I/R injury. As a result, Mdivi-1 attenuates neural mitochondria-mediated apoptosis, which is supported by the reductions in proapoptotic factors (e.g., AIF, Bax, cytochrome *c*, caspase-9, and caspase-3) as well as the increases in antiapoptotic factors (e.g., Bcl-2).

The main findings from evidence of included studies are synthesized as follow: (1) Mdivi-1 treatment could attenuate neural mitochondrial respiratory deficiency in the I/R-induced brain injury, as evidenced by the recovery of neural mitochondrial complex I-IV activities, the mitochondrial membrane potential, and the ATP production; (2) Mdivi-1 treatment could attenuate neural mitochondrial quality-control dysregulation in the I/R-induced brain injury, as evidenced by increases in the neural mitochondrial biogenesis regulators (PGC-1, TFAM, and NRF-1) and the level of mitochondrial DNA, as well as reductions in neural mitochondrial fission (Drp-1) and the neural mitophagy; (3) Mdivi-1 treatment could reduce neural mitochondria-mediated apoptosis in I/R-induced brain injury, as evidenced by reductions in mitochondria-mediated caspase-independent apoptotic factor (AIF), mitochondria-mediated caspase-dependent apoptotic upstream factor (Bax), and mitochondrial-mediated caspase-dependent apoptotic downstream factors (cytochrome *c*, caspase-9, and caspase-3) as well as increases in anti-apoptotic factor (Bcl-2). Taken together, Mdivi-1 treatment can improve neural mitochondrial respiratory function and neural mitochondrial quality-control, thereby reducing neural apoptosis through suppressing the mitochondria-mediated apoptotic pathways in I/R-induced brain injury (Figure 2). Therefore, we hypothesize that Mdivi-1 might be a promising therapeutic compound to attenuate I/R-induced brain injury after ischemic stroke.

The included study suggested that Mdivi-1 can normalize the activities of electron transport system, including complexes I–IV, attenuating neural mitochondrial respiratory deficiency in I/R-induced brain injury (Li et al., 2016). In support of this, a previous study showed that an intravenous injection of Mdivi-1 (1.2 mg/kg) enhanced the protein expressions of neural mitochondrial complex I in the brains of Sprague Dawley rats after subarachnoid hemorrhage (Fan et al., 2017). However, another study showed that, when neural mitochondrial respiration was impaired in neurons, Mdivi-1 (25–100 μM) inhibited the excessive activity of complex I to reduce neural mitochondria complex I-dependent oxidative stress (Bordt et al., 2017). It should be noted that neural oxidative stress is one of the main mechanisms in I/R-induced brain injury, which is partially caused by mitochondrial dysfunction and in turn promotes further neural mitochondrial damage (Stepien et al., 2017). We hypothesize that Mdivi-1 might act as a regulator in the activities of electron transport system to prevent the excessive release of reactive oxidative species in neurons and thereby reduce oxidative stress-induced damage to neural mitochondria in I/R-induced brain injury. Consistent with this hypothesis, the included study suggested that Mdivi-1 could reduce the oxidative stress induced by dysfunctional mitochondria in I/R neurons (Wang et al., 2014). Additionally, a previous study also showed that Mdivi-1 treatment attenuated hydrogen peroxide-induced mitochondrial dysfunction in PC12 cells (Song et al., 2019). Mdivi-1 appears to have dual effects that could interrupt the interdependence between neural mitochondrial respiratory deficiency and neural oxidative stress in I/R-induced brain injury after ischemic stroke.

Under physiological condition, mitochondrial membrane potential (MMP) is involved in Krebs cycle, which is the energy storage form used to synthesis ATP (Zorova et al., 2018). In addition, the normal MMP was proven to be a key factor in the removal of dysfunctional mitochondria in cells (Zorova et al., 2018). In ischemic stroke, the depolarization of MMP is promoted, leading to ATP depletion and neural cell death (Hu et al., 2018; Zorova et al., 2018). The review provided that Mdivi-1 can normalize MMP and thereby enhance ATP production both *in vivo* and *in vitro* (Zhao et al., 2014; Li et al., 2016; Zhou et al., 2017; Wang et al., 2018; Huang et al., 2021), suggesting that Mdivi-1 treatment can attenuate neural mitochondrial respiratory deficiency as well as maintain the number of healthy neural mitochondria in I/R-induced brain injury. Of note, MPP is produced by the activities of neural mitochondrial complexes I, III, and IV (Zorova et al., 2018). Thus, the reviewed finding suggested that Mdivi-1 might restore MMP by normalizing the activities of neural mitochondrial complexes as mentioned above.

Previous studies reported that neural mitochondrial biogenesis regulators (e.g., PGC-1α, TFAM, NRF-1, and NRF-2) were upregulated as a compensatory response to energy demand and reductions in neural mitochondrial content in I/R-induced brain injury (Ma et al., 2016; Carinci et al., 2021). Therefore, neural mitochondrial biogenesis was considered the target for therapeutic agents in I/R-induced brain injury after stroke (Carinci et al., 2021). In addition, the upregulation of PGC-1α has been shown to protect neurons against oxidative stress (Yang et al., 2018; Carinci et al., 2021). The included studies showed that the protein levels of mitochondrial biogenesis regulators (i.e., PGC-1α, TFAM, and NRF-1) as well as the level of mitochondrial DNA were further increased by Mdivi-1 treatment (Ma et al., 2016; Huang et al., 2021). Supportively, a previous study showed that treatment with Mdivi-1 (25 and 75 μM) could increase the protein levels of PGC-1α, NRF-1, and NRF-2 in N2a cells with Drp1 RNA silenced (Manczak et al., 2019). Collectively, the current evidence indicates that the enhancement of neural mitochondrial biogenesis might be one of the therapeutic effects of Mdivi-1 treatment on I/R-induced brain injury after ischemic stroke. We also hypothesize that PGC-1α activation is the neural mitochondria-related mechanism by which Mdivi-1 attenuates oxidative stress in I/R-induced brain injury after ischemic stroke.

Drp-1 inhibition is the typical characteristic of Mdivi-1, which was repeatedly confirmed in I/R-induced brain injury in the included studies (Zhang et al., 2013; Wang et al., 2014; Li et al., 2015, 2016; Chuang et al., 2016; Ma et al., 2016; Zhou et al., 2017). Likewise, a previous study showed that treatment with Mdivi-1 (1.2 mg/kg, intravenous injection) significantly reduced neural mitochondrial fission in rats with subarachnoid hemorrhage (Wu et al., 2017). However, another previous study showed that Mdivi-1 could inhibit neural mitochondrial fission in NMDA-treated neurons with Drp-1 knockdown, suggesting that the fission-inhibitory effects of Mdivi-1 might not completely depend on Drp-1 (Ruiz et al., 2018). Therefore, the explanations for therapeutic effects of Mdivi-1 in I/R-induced brain injury are not only based on the Drp-1 inhibition.

Regarding the effects of Mdivi-1 on neural mitochondrial fusion, the included studies showed that Mdivi-1 only increased neural outer membrane mitochondrial fusion (e.g., Mfn1 factor) but did not affect the neural inner membrane mitochondrial fusion (e.g., Opa1 factor) in I/R-induced brain injury (Ma et al., 2016; Huang et al., 2021). The effects of Mdivi-1 on neural mitochondrial fusion were also controversial among previous studies. A previous study showed that Mdivi-1 (20 mg/kg, i.p.) did not change the protein levels of two typical neural mitochondrial fusion factors (Opa1 and Mfn1) in kainic acid-damaged hippocampal cells of 4-week old mice (Kim et al., 2016). However, one study reported that Mdivi-1 treatment (1.2 mg/kg, i.p.) increased the protein level of Opa1 against lipopolysaccharide-induced brain damage (Deng et al., 2018). Another study showed that Mdivi-1 treatment (25 and 75 μM) also enhanced the levels of fusion proteins, including Mfn1, Mfn2, and Opa1 in N2a cells with Drp-1 RNA silenced (Manczak et al., 2019). This discrepancy might partially be explained by the differences in the animal models and Mdivi-1 administrations among studies. Due to lack of data, the current systematic review cannot indicate the effects of Mdivi-1 on neural mitochondrial fusion in I/R injury after ischemic stroke. It should be noted that fusion and fission of neural mitochondria were suggested to be important in determining the extent of ischemic injury after stroke (Carinci et al., 2021). Because Mdivi-1 could attenuate neural mitochondrial fission and might not induce negative impacts on neural mitochondrial fusion as observed in the included studies, Mdivi-1 could reduce the extent of I/R-induced brain injury after ischemic stroke.

In the included studies, Mdivi-1 treatment reduced the level of dysfunctional mitochondrial detectors (i.e., PINK1 and Parkin) and maintained the number of healthy mitochondria, suggesting that Mdivi-1 might attenuate the impairment of neural mitochondrial functions to reduce the neural mitophagy in I/R-induced brain injury (Zhang et al., 2014; Huang et al., 2021). However, it is unclear whether the mitophagy-inhibitory effects of Mdivi-1 is the therapeutic effect or not because the roles of neural mitophagy varies in ischemic stroke (Shao et al., 2020). Accumulated evidence has shown that neural mitophagy is both beneficial and harmful, partially depending on the severity and stage of ischemic injury, which interact with neural apoptosis (Shao et al., 2020). Therefore, further studies should compare the effects of Mdivi-1 on mitophagy and neural apoptosis in any stages of ischemic stroke to comprehensively clarify the therapeutic effects of Mdivi-1.

Our review suggested that Mdivi-1 can inactivate mitochondria-mediated apoptotic pathways, which is supported by the reduced levels of proapoptotic factors, including AIF, Bax, cytochrome *c*, caspase-9, and caspase-3 and increased levels of antiapoptotic factor (Bcl-2) (Zhang et al., 2013; Wang et al., 2014, 2018; Zhao et al., 2014; Li et al., 2015, 2016; Chuang et al., 2016; Ma et al., 2016; Zhou et al., 2017). Consistently, one study showed that pretreatment with 50 μM Mdivi-1 reduced the proportion of apoptotic cells as well as the levels of Bax, cytosolic cytochrome *c*, and caspase-3 in glutamate-damaged cortical neurons *in vitro*, implying that Mdivi-1 might reduce neural apoptosis in the brain against the overexpression of glutamate, a primary mechanism of I/R-induced brain injury after ischemic stroke (Zhou et al., 2018). Likewise, previous studies showed that the intravenous injection of Mdivi-1 (1.2 mg/kg) could reduce the TUNEL-apoptotic positive cells as well as the protein levels of Bax, cytochrome *c*, and cleaved caspase-3 in the brains of Sprague Dawley rats after subarachnoid hemorrhage (Fan et al., 2017; Wu et al., 2017). One possible explanation for antiapoptotic effects of Mdivi-1 is that Mdivi-1 could attenuate the neural mitochondrial permeability impairment in stroke, preventing the translocation of Bax into neural mitochondria and thus inactivating neural mitochondria-related apoptotic pathways (Zhao et al., 2014). Mdivi-1 also reduced mPTP opening in the inner mitochondrial membrane, thereby inactivate the release of AIF and cytochrome *c* from neural mitochondria to neural cytosol (Zhou et al., 2017). Of note, mitochondria-mediated apoptotic pathways are typical activated after ischemic stroke, occurring when neural mitochondria functions are impaired and cannot manage the damage of I/R-induced brain injury (Guan et al., 2018; Shao et al., 2020). Thus, the current evidence indicates that Mdivi-1 might suppress mitochondria-related apoptotic pathways by improving the neural mitochondrial functions, thereby protecting the brain against I/R-induced brain injury after ischemic stroke.

### Study Quality Evaluation

In our systematic review, the quality of the included *in vivo* studies was evaluated by the CAMARADES checklist, which is commonly used in animal studies (Auboire et al., 2018). As a commonplace characteristic of preclinical research, the included *in vivo* studies herein did not calculate simple size (*n* = 12, 100%), conduct the allocation concealment (*n* = 12, 100%) or assess outcome in a blinded manner (*n* = 11, 92%). However, selection bias was reduced because all studies followed the regulatory requirements and clearly described the animal models and surgery procedures. Moreover, major studies (*n* = 6, 75%) randomized animals into experimental groups. In addition, the studies were published in peer-reviewed journals and declared no conflicts of interest, thereby minimizing the reporting bias and publication bias for our review findings. Regarding *in vitro* studies, the TOXRTOOL checklist has been used in many studies to strictly evaluate the quality of *in vitro* studies (Schneider et al., 2009). The included *in vitro* studies met most requirements of the checklist, meaning that the results from those studies were considered reliable. The findings from both the *in vivo* and *in vitro* included studies were consistent, suggesting that our reviewed findings might be reasonable but not ultimately conclusive due to a lack of data; thus, they need to be supported by further studies.

### Limitations

Several limitations need to be considered in this systematic review. First, we included evidence from full-text English articles but not from papers in other languages, conference abstracts or locally published articles, which might have increased publication bias for our reviewed findings. Second, the included studies used various models of ischemic stroke that increased biases to summarize the benefits of Mdivi-1 on neural mitochondrial functions and neural mitochondria-mediated apoptosis. Third, the dosage and timing of Mdivi-1 treatment were diverse, and only two studies compared several doses of Mdivi-1 in ischemic stroke, restricting the comprehensive provision of the effects of Mdivi-1. Fourth, most studies used male animals, and no study compared the effects of Mdivi-1 on neural mitochondrial functions and neural apoptosis in the two sexes. Therefore, the review cannot provide evidence of sex-specific mitochondria-related mechanisms underlying the effects of Mdivi-1. Finally, this review only provided the effects of Mdivi-1 on neural mitochondrial functions as well as neural mitochondria-related apoptosis, but it did not provide cause-effects for why Mdivi-1 induces those benefits.

### Implications for Further Research

Previous studies have shown that neural mitochondrial dysfunction could activate neuroinflammation and further promote neural cell deaths in I/R-induced brain injury (Carinci et al., 2021). Ischemic injury might activate the mPTP to open, releasing mitochondrial proinflammatory factors, including ATP, AIF, and HSP60, from neural mitochondria to extracellular spaces and then interacting with receptors in inflammatory cells, such as astrocytes and microglia, to increase proinflammatory factors (e.g., tumor necrosis factor-alpha, interleukin-1, and interleukin-18) (Galluzzi et al., 2012; Jung and Seong, 2021). These proinflammatory factors in turn activate caspases to induce inflammatory programmed cell death (Jung and Seong, 2021). Our review provided that Mdivi-1 can enhance neural mitochondrial functions, thereby posing a hypothesis that Mdivi-1 attenuates neuroinflammation in I/R-induced brain injury after ischemic stroke. Supportively, one study showed that Mdivi-1 (1.2 mg/kg) injection after subarachnoid hemorrhage inactivated nuclear translocation of NF-κB and thereby reduced the levels of TNF-α, IL-6, and IL-1ß in ischemic-induced brain damage in MCAO rat models (Fan et al., 2017). Another study showed that Mdivi-1 treatment (25 mg/kg, i.p.) reduced neuroinflammatory T-cells (Th1 and Th17) in the brains of an autoimmune encephalomyelitis mouse model (Li et al., 2019). To comprehensively evaluate the therapeutic characteristics of Mdivi-1 in ischemic stroke, further studies are required to investigate the effects of Mdivi-1 on the relationship between mitochondrial dysfunction and neuroinflammation in ischemic brain injury.

Previous studies have shown that ischemic injury also induces neural apoptosis in the brain by promoting the extrinsic-mediated apoptotic pathway, which is accompanied by the augmentation of cellular calcium influx (Carinci et al., 2021). One study showed that Mdivi-1 could control the neural mitochondrial calcium concentration, thereby attenuating neural mitochondrial dysfunction and neural apoptosis in NMDA-treated neurons (Ruiz et al., 2018). However, to the best of our knowledge, there is no evidence regarding the effects of Midi-1 on extrinsic apoptotic pathways in the brain after ischemic stroke. This issue should be addressed by further studies to provide additional evidence on the potential effects of Mdivi-1 on neural apoptosis in ischemic stroke.

The findings from previous studies suggested that Mdivi-1 showed mitochondria-related therapeutic effects on ischemic stroke as well as other disorders in the central nervous system (Fan et al., 2017; Li et al., 2019; Song et al., 2019). Regarding neurodegenerative disorders that might be promoted after ischemic stroke such as Alzheimer’s disease (Vijayan and Reddy, 2016) and Parkinsonism (Harnod et al., 2020), Mdivi-1 has been proven to increase mitochondrial length and ATP level, as well as reduce the mitochondrial depolarization of Aβ-treated hippocampal cells in Alzheimer’s disease (Baek et al., 2017). Likewise, treatment with Mdivi-1 could also enhance mitochondrial respiration and reduce neural mitochondrial fragmentation in the Parkinsonian’s rat model (Bido et al., 2017). Moreover, Mdivi-1 has been shown to have a high capacity to cross the brain-blood barrier, supporting its potential therapeutic effects on the brain (Cui et al., 2016). However, alongside therapeutic benefits mentioned above, the off-target effects of Mdivi-1 has been reported. A previous study showed that treatment with 50 μM Mdivi-1 caused mitochondrial depolarization, oxidative stress, and apoptosis in oligodendrocytes with excitotoxicity (Ruiz et al., 2020), suggesting the complicated effects of Mdivi-1 on mitochondrial functions which might be influenced by many factors, including dosage and targeted cells. Currently, the off-target effects of Mdivi-1 on neural cells under I/R injury have not been explored. In addition, there are no preclinical data on side effects and the optimal administration of Mdivi-1 (dose, time window, injection method) for ischemic stroke, making it impossible to apply Mdivi-1 treatment in humans and thus requiring additional data from further studies.

## Conclusion

Our systematic review summarized the current evidence from both *in vivo* and *in vitro* studies to evaluate the therapeutic effects of Mdivi-1 on neural mitochondrial functions and neural mitochondria-related apoptosis in I/R injury after ischemic stroke. From the included studies with several ischemic stroke models, our review suggests that Mdivi-1 treatment might attenuate neural mitochondrial respiratory deficiency as well as neural mitochondrial quality-control dysregulation and thus reduce neural mitochondria-mediated apoptosis. These findings imply that Mdivi-1 might be a potential target for therapy in ischemic stroke. However, the use of various models, an array of protocols as well as the lack of data made the findings inconclusive, despite their reliability. Therefore, further studies are required to support the neuroprotective effects of Mdivi-1 in I/R-induced brain injury, including neural mitochondrial fusion and the mitochondria-related anti-inflammatory mechanisms in both acute and chronic stages of ischemic stroke. In addition, the optimal administration, toxin effects and side-effects of Mdivi-1 treatment should be clarified to find a way to apply Mdivi-1 in clinical trials.

## Data Availability Statement

The original contributions presented in the study are included in the article/supplementary material, further inquiries can be directed to the corresponding author.

## Author Contributions

NTN, S-YX, and S-DL contributed to the conceptualization and edited and revised the manuscript. NTN, JX, and S-DL contributed to the methodology. NTN, QL, YL, and S-YX contributed to the collection, synthesis, and interpretation of the data. NTN drafted the manuscript. All authors approved the final version of the manuscript.

## Conflict of Interest

The authors declare that the research was conducted in the absence of any commercial or financial relationships that could be construed as a potential conflict of interest.

## Publisher’s Note

All claims expressed in this article are solely those of the authors and do not necessarily represent those of their affiliated organizations, or those of the publisher, the editors and the reviewers. Any product that may be evaluated in this article, or claim that may be made by its manufacturer, is not guaranteed or endorsed by the publisher.

## References

[B1] Andrabi S. S. Parvez S. Tabassum H. (2020). Ischemic stroke and mitochondria: mechanisms and targets. *Protoplasma* 257 335–343. 10.1007/s00709-019-01439-2 31612315

[B2] Anzell A. R. Maizy R. Przyklenk K. Sanderson T. H. (2018). Mitochondrial quality control and disease: insights into ischemia-reperfusion injury. *Mol. Neurobiol.* 55 2547–2564. 10.1007/s12035-017-0503-9 28401475 PMC5636654

[B3] Auboire L. Sennoga C. A. Hyvelin J.-M. Ossant F. Escoffre J.-M. Tranquart F. (2018). Quality assessment of the studies using the collaborative approach to meta-analysis and review of Animal Data from Experimental Studies (CAMARADES) checklist items. *PLoS One.* 10.1371/journal.pone.0191788.t007

[B4] Baek S. H. Park S. J. Jeong J. I. Kim S. H. Han J. Kyung J. W. (2017). Inhibition of Drp1 ameliorates synaptic depression, abeta deposition, and cognitive impairment in an Alzheimer’s disease model. *J. Neurosci.* 37 5099–5110. 10.1523/JNEUROSCI.2385-16.2017 28432138 PMC6596467

[B5] Bido S. Soria F. N. Fan R. Z. Bezard E. Tieu K. (2017). Mitochondrial division inhibitor-1 is neuroprotective in the A53T-alpha-synuclein rat model of Parkinson’s disease. *Sci. Rep.* 7:7495. 10.1038/s41598-017-07181-0 28790323 PMC5548731

[B6] Bordt E. A. Clerc P. Roelofs B. A. Saladino A. J. Tretter L. dam-Vizi V. A. (2017). The putative Drp1 inhibitor mdivi-1 is a reversible mitochondrial Complex I inhibitor that modulates reactive oxygen species. *Dev. Cell* 40 583.e6–594.e6. 10.1016/j.devcel.2017.02.020 28350990 PMC5398851

[B7] Broughton B. R. S. Reutens D. C. Sobey C. G. (2009). Apoptotic mechanisms after cerebral ischemia. *Stroke* 40 e331–e339. 19182083 10.1161/STROKEAHA.108.531632

[B8] Carinci M. Vezzani B. Patergnani S. Ludewig P. Lessmann K. Magnus T. (2021). Different roles of mitochondria in cell death and inflammation: focusing on mitochondrial quality control in ischemic stroke and reperfusion. *Biomedicines* 9:169. 10.3390/biomedicines9020169 33572080 PMC7914955

[B9] Cassidy-Stone A. Chipuk J. E. Ingerman E. Song C. Yoo C. Kuwana T. (2008). Chemical inhibition of the mitochondrial division dynamin reveals its role in Bax/Bak-dependent mitochondrial outer membrane permeabilization. *Dev. Cell* 14 193–204. 10.1016/j.devcel.2007.11.019 18267088 PMC2267902

[B10] Chuang Y. C. Lin T. K. Yang D. I. Yang J. L. Liou C. W. Chen S. D. (2016). Peroxisome proliferator-activated receptor-gamma dependent pathway reduces the phosphorylation of dynamin-related protein 1 and ameliorates hippocampal injury induced by global ischemia in rats. *J. Biomed. Sci.* 23:44. 10.1186/s12929-016-0262-3 27175924 PMC4865999

[B11] Cui M. Ding H. Chen F. Zhao Y. Yang Q. Dong Q. (2016). Mdivi-1 protects against ischemic brain injury via elevating extracellular adenosine in a cAMP/CREB-CD39-dependent manner. *Mol. Neurobiol.* 53 240–253. 10.1007/s12035-014-9002-4 25428621

[B12] Deng S. Ai Y. Gong H. Feng Q. Li X. Chen C. (2018). Mitochondrial dynamics and protective effects of a mitochondrial division inhibitor, Mdivi-1, in lipopolysaccharide-induced brain damage. *Biochem. Biophys. Res. Commun.* 496 865–871. 10.1016/j.bbrc.2018.01.136 29395086

[B13] Duan C. Wang L. Zhang J. Xiang X. Wu Y. Zhang Z. (2020). Mdivi-1 attenuates oxidative stress and exerts vascular protection in ischemic/hypoxic injury by a mechanism independent of Drp1 GTPase activity. *Redox Biol.* 37:101706. 10.1016/j.redox.2020.101706 32911435 PMC7490562

[B14] Fan L. F. He P. Y. Peng Y. C. Du Q. H. Ma Y. J. Jin J. X. (2017). Mdivi-1 ameliorates early brain injury after subarachnoid hemorrhage via the suppression of inflammation-related blood-brain barrier disruption and endoplasmic reticulum stress-based apoptosis. *Free Radic. Biol. Med.* 112 336–349. 10.1016/j.freeradbiomed.2017.08.003 28790012

[B15] Galkin A. (2019). Brain Ischemia/Reperfusion injury and mitochondrial complex I damage. *Biochemistry (Mosc)* 84 1411–1423. 10.1134/s0006297919110154 31760927 PMC12994863

[B16] Galluzzi L. Kepp O. Kroemer G. (2012). Mitochondria: master regulators of danger signalling. *Nat. Rev. Mol. Cell Biol.* 13 780–788. 10.1038/nrm3479 23175281

[B17] Guan R. Zou W. Dai X. Yu X. Liu H. Chen Q. (2018). Mitophagy, a potential therapeutic target for stroke. *J. Biomed. Sci.* 25:87. 10.1186/s12929-018-0487-4 30501621 PMC6271612

[B18] Harnod D. Harnod T. Lin C. L. Hsu C. Y. Kao C. H. (2020). Poststroke Parkinsonism associates with an increased mortality risk in patients. *Ann. Transl. Med.* 8:471. 10.21037/atm.2020.03.90 32395515 PMC7210154

[B19] He Z. Ning N. Zhou Q. Khoshnam S. E. Farzaneh M. (2020). Mitochondria as a therapeutic target for ischemic stroke. *Free Radic. Biol. Med.* 146 45–58. 10.1016/j.freeradbiomed.2019.11.005 31704373

[B20] Hu Q. Tang J. Manaenko A. Lu J. Liu F. (2018). Mitochondria in ischemic stroke: new insight and implications. *Aging Dis.* 9 924–937. 30271667 10.14336/AD.2017.1126 PMC6147588

[B21] Huang Y. Gao X. Zhou X. Zhang Y. Tan Z. Zhu S. (2021). Remote ischemic postconditioning inhibited mitophagy to achieve neuroprotective effects in the rat model of cardiac arrest. *Neurochem. Res.* 46 573–583. 10.1007/s11064-020-03193-x 33409854

[B22] Jung K.-H. Seong S.-Y. (2021). Role of inflammasomes in neuroinflammation after ischemic stroke. *Encephalitis* 1 89–97. 10.47936/encephalitis.2021.00073 PMC10295893 37470048

[B23] Kim H. Lee J. Y. Park K. J. Kim W.-H. Roh G. S. (2016). A mitochondrial division inhibitor, Mdivi-1, inhibits mitochondrial fragmentation and attenuates kainic acid-induced hippocampal cell death. *BMC Neurosci.* 17:33. 10.1186/s12868-016-0270-y 27287829 PMC4902937

[B24] Li Y. Wang M. Wang S. (2016). Effect of inhibiting mitochondrial fission on energy metabolism in rat hippocampal neurons during ischemia/reperfusion injury. *Neurol. Res.* 38 1027–1034. 10.1080/01616412.2016.1215050 30919739

[B25] Li Y. Wang P. Wei J. Fan R. Zuo Y. Shi M. (2015). Inhibition of Drp1 by Mdivi-1 attenuates cerebral ischemic injury via inhibition of the mitochondria-dependent apoptotic pathway after cardiac arrest. *Neuroscience* 311 67–74. 10.1016/j.neuroscience.2015.10.020 26477985

[B26] Li Y.-H. Xu F. Thome R. Guo M.-F. Sun M.-L. Song G.-B. (2019). Mdivi-1, a mitochondrial fission inhibitor, modulates T helper cells and suppresses the development of experimental autoimmune encephalomyelitis. *J. Neuroinflamm.* 16:149. 10.1186/s12974-019-1542-0 31324254 PMC6642537

[B27] Ma X. Xie Y. Chen Y. Han B. Li J. Qi S. (2016). Post-ischemia mdivi-1 treatment protects against ischemia/reperfusion-induced brain injury in a rat model. *Neurosci. Lett.* 632 23–32. 10.1016/j.neulet.2016.08.026 27542342

[B28] Manczak M. Kandimalla R. Yin X. Reddy P. H. (2019). Mitochondrial division inhibitor 1 reduces dynamin-related protein 1 and mitochondrial fission activity. *Hum. Mol. Genet.* 28 177–199. 10.1093/hmg/ddy335 30239719 PMC6322070

[B29] Ouyang Y. B. Giffard R. G. (2014). MicroRNAs affect BCL-2 family proteins in the setting of cerebral ischemia. *Neurochem. Int.* 77 2–8. 10.1016/j.neuint.2013.12.006 24373752 PMC4071131

[B30] Page M. J. McKenzie J. E. Bossuyt P. M. Boutron I. Hoffmann T. C. Mulrow C. D. (2021). The PRISMA 2020 statement: an updated guideline for reporting systematic reviews. *BMJ* 372:n71. 10.1136/bmj.n71 PMC8005924 33782057

[B31] Roy-O’Reilly M. McCullough L. D. (2018). Age and sex are critical factors in ischemic stroke pathology. *Endocrinology* 159 3120–3131. 10.1210/en.2018-00465 30010821 PMC6963709

[B32] Ruiz A. Alberdi E. Matute C. (2018). Mitochondrial division inhibitor 1 (mdivi-1) protects neurons against excitotoxicity through the modulation of mitochondrial function and intracellular Ca2+ signaling. *Front. Mol. Neurosci.* 11:3. 10.3389/fnmol.2018.00003 29386996 PMC5776080

[B33] Ruiz A. Quintela-Lopez T. Sanchez-Gomez M. V. Gaminde-Blasco A. Alberdi E. Matute C. (2020). Mitochondrial division inhibitor 1 disrupts oligodendrocyte Ca(2+) homeostasis and mitochondrial function. *Glia* 68 1743–1756. 10.1002/glia.23802 32060978

[B34] Schneider K. Schwarz M. Burkholder I. Kopp-Schneider A. Edler L. Kinsner-Ovaskainen A. (2009). “ToxRTool”, a new tool to assess the reliability of toxicological data. *Toxicol. Lett.* 189 138–144. 10.1016/j.toxlet.2009.05.013 19477248

[B35] Sekerdag E. Solaroglu I. Gursoy-Ozdemir Y. (2018). Cell death mechanisms in stroke and novel molecular and cellular treatment options. *Curr. Neuropharmacol.* 16 1396–1415. 10.2174/1570159X16666180302115544 29512465 PMC6251049

[B36] Shao Z. Dou S. Zhu J. Wang H. Xu D. Wang C. (2020). The role of mitophagy in ischemic stroke. *Front. Neurol.* 11:608610. 10.3389/fneur.2020.608610 33424757 PMC7793663

[B37] Song Y. Li T. Liu Z. Xu Z. Zhang Z. Chi L. (2019). Inhibition of Drp1 after traumatic brain injury provides brain protection and improves behavioral performance in rats. *Chem.-Biol. Interact.* 304 173–185. 10.1016/j.cbi.2019.03.013 30894316

[B38] Stepien K. M. Heaton R. Rankin S. Murphy A. Bentley J. Sexton D. (2017). Evidence of oxidative stress and secondary mitochondrial dysfunction in metabolic and non-metabolic disorders. *J. Clin. Med.* 6:71. 10.3390/jcm6070071 28753922 PMC5532579

[B39] Uzdensky A. B. (2019). Apoptosis regulation in the penumbra after ischemic stroke: expression of pro- and antiapoptotic proteins. *Apoptosis* 24 687–702. 10.1007/s10495-019-01556-6 31256300

[B40] Vijayan M. Reddy P. H. (2016). Stroke, Vascular Dementia, and Alzheimer’s disease: molecular links. *J. Alzheimers Dis.* 54 427–443. 10.3233/jad-160527 27567871 PMC5793908

[B41] Wang J. Wang P. Li S. Wang S. Li Y. Liang N. (2014). Mdivi-1 prevents apoptosis induced by ischemia-reperfusion injury in primary hippocampal cells via inhibition of reactive oxygen species-activated mitochondrial pathway. *J. Stroke Cerebrovasc. Dis.* 23 1491–1499. 10.1016/j.jstrokecerebrovasdis.2013.12.021 24774441

[B42] Wang P. Li Y. Yang Z. Yu T. Zheng G. Fang X. (2018). Inhibition of dynamin-related protein 1 has neuroprotective effect comparable with therapeutic hypothermia in a rat model of cardiac arrest. *Transl. Res.* 194 68–78. 10.1016/j.trsl.2018.01.002 29351829

[B43] Wu P. Li Y. Zhu S. Wang C. Dai J. Zhang G. (2017). Mdivi-1 alleviates early brain injury after experimental subarachnoid hemorrhage in rats, possibly via inhibition of Drp1-activated mitochondrial fission and oxidative stress. *Neurochem. Res.* 42 1449–1458. 10.1007/s11064-017-2201-4 28210956

[B44] Yang J. L. Mukda S. Chen S. D. (2018). Diverse roles of mitochondria in ischemic stroke. *Redox Biol.* 16 263–275. 10.1016/j.redox.2018.03.002 29549824 PMC5854930

[B45] Zhang N. Wang S. Li Y. Che L. Zhao Q. (2013). A selective inhibitor of Drp1, mdivi-1, acts against cerebral ischemia/reperfusion injury via an anti-apoptotic pathway in rats. *Neurosci. Lett.* 535 104–109. 10.1016/j.neulet.2012.12.049 23313133

[B46] Zhang X. Yan H. Yuan Y. Gao J. Shen Z. Cheng Y. (2014). Cerebral ischemia-reperfusion-induced autophagy protects against neuronal injury by mitochondrial clearance. *Autophagy* 9 1321–1333. 10.4161/auto.25132 23800795

[B47] Zhang Z. Yang X. Zhang S. Ma X. Kong J. (2007). BNIP3 upregulation and EndoG translocation in delayed neuronal death in stroke and in hypoxia. *Stroke* 38 1606–1613. 10.1161/STROKEAHA.106.475129 17379825

[B48] Zhao Y. X. Cui M. Chen S. F. Dong Q. Liu X. Y. (2014). Amelioration of ischemic mitochondrial injury and Bax-dependent outer membrane permeabilization by Mdivi-1. *CNS Neurosci. Ther.* 20 528–538. 10.1111/cns.12266 24712408 PMC6493009

[B49] Zhou K. Yang H. Y. Tang P. Y. Liu W. Luo Y. J. Lv B. (2018). Mitochondrial division inhibitor 1 protects cortical neurons from excitotoxicity: a mechanistic pathway. *Neural Regen. Res.* 13 1552–1560. 10.4103/1673-5374.235299 30127115 PMC6126130

[B50] Zhou X. Wang H.-Y. Wu B. Cheng C.-Y. Xiao W. Wang Z.-Z. (2017). Ginkgolide K attenuates neuronal injury after ischemic stroke by inhibiting mitochondrial fission and GSK-3β-dependent increases in mitochondrial membrane permeability. *Oncotarget* 8 44682–44693. 10.18632/oncotarget.17967 28591721 PMC5546510

[B51] Zorova L. D. Popkov V. A. Plotnikov E. Y. Silachev D. N. Pevzner I. B. Jankauskas S. S. (2018). Mitochondrial membrane potential. *Anal. Biochem.* 552 50–59. 28711444 10.1016/j.ab.2017.07.009 PMC5792320

